# Physical models of bacterial chromosomes

**DOI:** 10.1111/mmi.15257

**Published:** 2024-04-05

**Authors:** Janni Harju, Chase P. Broedersz

**Affiliations:** ^1^ Department of Physics and Astronomy Vrije Universiteit Amsterdam Amsterdam The Netherlands; ^2^ Department of Physics, Arnold Sommerfeld Center for Theoretical Physics and Center for NanoScience Ludwig‐Maximilian‐University Munich Munich Germany

**Keywords:** Bacterial chromosomes, Data‐driven modeling, Loop extrusion, Maximum Entropy

## Abstract

The interplay between bacterial chromosome organization and functions such as transcription and replication can be studied in increasing detail using novel experimental techniques. Interpreting the resulting quantitative data, however, can be theoretically challenging. In this minireview, we discuss how connecting experimental observations to biophysical theory and modeling can give rise to new insights on bacterial chromosome organization. We consider three flavors of models of increasing complexity: simple polymer models that explore how physical constraints, such as confinement or plectoneme branching, can affect bacterial chromosome organization; bottom‐up mechanistic models that connect these constraints to their underlying causes, for instance, chromosome compaction to macromolecular crowding, or supercoiling to transcription; and finally, data‐driven methods for inferring interpretable and quantitative models directly from complex experimental data. Using recent examples, we discuss how biophysical models can both deepen our understanding of how bacterial chromosomes are structured and give rise to novel predictions about bacterial chromosome organization.

## Graphical abstract

Connecting experimental observations to biophysical models can give rise to new insights on bacterial chromosome organization. Simple polymer models explore how physical constraints affect bacterial chromosome organization; bottom‐up mechanistic models connect these constraints to their underlying causes; and data‐driven models are inferred directly from complex experimental data.

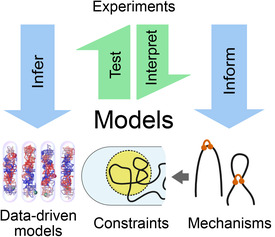

## 1 INTRODUCTION

The genome of many bacterial species is contained in a single circular chromosome, which is compressed by orders of magnitude into the bacterial cell. Microscopy studies have revealed that various factors, such as transcription, nucleoid‐associated proteins (NAPs), supercoiling, loop‐extrusion by SMC complexes, and replication shape bacterial chromosome organization (Dame et al., 2020; Gogou et al., 2021; Lioy et al., 2021; Yáñez‐Cuna & Koszul, 2023). However, since microscopy experiments cannot resolve the full 3D conformation of a bacterial chromosome, the effects of pharmacological perturbations on chromosome organization are often only explored indirectly, for instance by observing how they affect chromosome compaction or segregation. Alternatively, chromosome organization can be studied using sequencing‐based methods, including chromosome conformation capture experiments such as Hi‐C (Le et al., 2013). Hi‐C experiments measure how often pairs of loci are spatially proximate, or “in contact,” averaged over a population of cells. Although interpreting Hi‐C data remains challenging, these and other high‐resolution quantitative data open new avenues to address old, yet unanswered questions: how are bacterial chromosomes organized across scales? how do various biological mechanisms control this organization? and, how does chromosome organization facilitate biological functions?

The recent surge in experimental techniques to quantitatively probe bacterial chromosome organization poses new and exciting challenges for biophysical modeling. Theoretical and computational biophysical models use concepts from polymer physics to explore how different mechanisms, such as macromolecular crowding (Polson & Kerry, 2018; Rivas & Minton, 2016) or bridging by NAPs (Amemiya et al., 2021; Dame et al., 2020), can affect chromosome organization and dynamics. This minireview focuses on recent models for bacterial chromosome organization, grouped according to their underlying modeling approach, and ordered by increasing complexity. First, we discuss polymer models that study *how geometrical and topological constraints affect bacterial chromosomes*, such as how cellular confinement or polymer branching influences chromosome conformation and dynamics. Second, we explore bottom‐up models, which study *how chromosome organization emerges from microscopic mechanisms*, like how loop‐extrusion by SMC complexes (~50 nm) (Banigan & Mirny, 2020; Fudenberg et al., 2017) can organize chromosomes at the nucleoid scale (~1 μm). Finally, we consider data‐driven approaches, which seek to *infer a model for chromosome organization, given experimental data*, providing a physical interpretation for Hi‐C maps. We discuss various benefits and limitations of these different modeling approaches, explore future modeling opportunities and challenges, and summarize key insights into bacterial chromosome organization gained via biophysical modeling.

## 2 GEOMETRIC AND TOPOLOGICAL CONSTRAINTS DETERMINE CHROMOSOME ORGANIZATION

**FIGURE 1:**
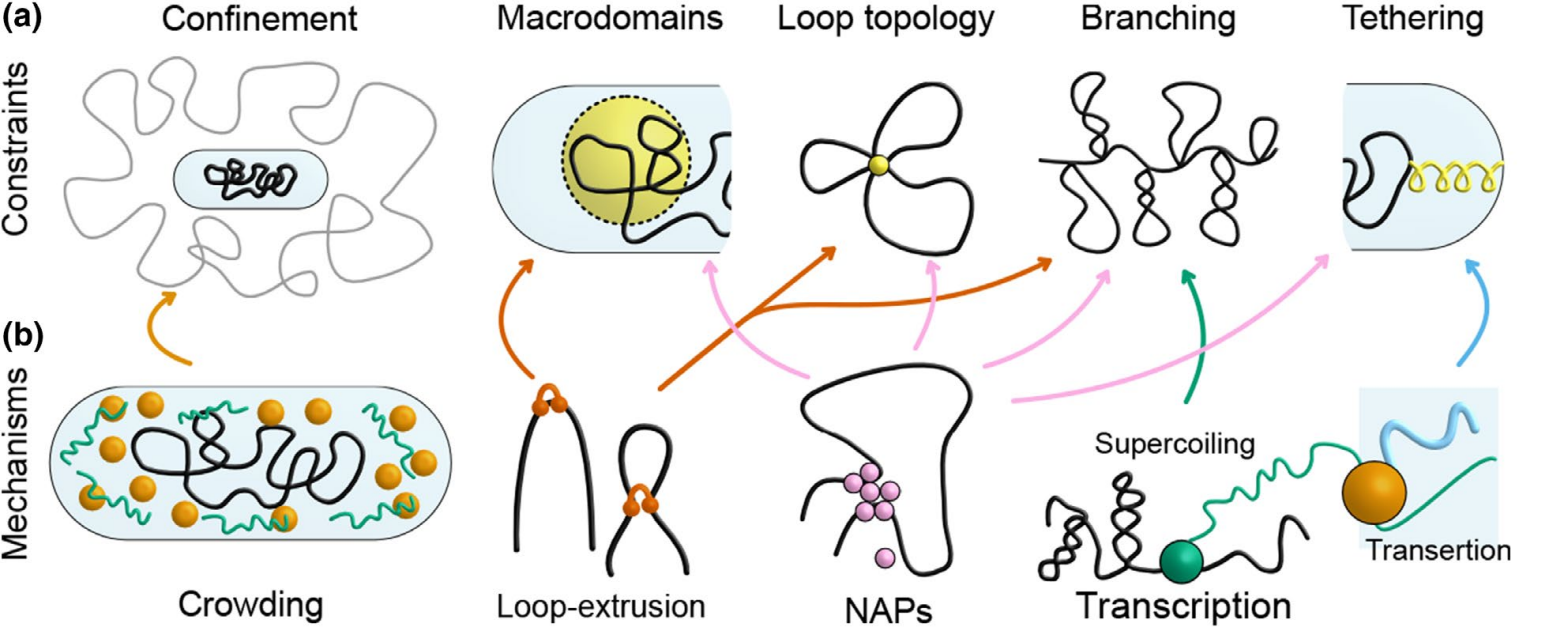
Constraint‐based and bottom‐up models. (a) Examples of constraint‐based models. Left to right: Polymer confinement can give rise to effects such as entropic segregation (Jun & Mulder, 2006). Constraining chromosomal regions corresponding to *E. coli* macrodomains to subvolumes of the nucleoid can give rise to order and chromosome segregation (Junier et al., 2014). Fixed loop topologies can orient bacterial chromosomes and enhance their segregation (Mitra et al., 2022b). Feather‐boa models show that branching can affect chromosome compaction, segregation, and dynamics. Constraining origins of replication or other loci by tethering them to the cell membrane can give rise to linear ordering of the chromosome (Buenemann & Lenz, 2010, 2011). (b) Examples of bottom‐up mechanisms. Left to right: Macromolecular crowding due to RNA, ribosomes, and other large molecules can compact bacterial chromosomes (Rivas & Minton, 2016). Loop‐extrusion by MukBEF can contribute to macrodomain formation in *E. coli* (Lioy et al., 2018), whereas condensin can tie together the chromosomal arms in *C. crescentus* and *B. subtilis* (Le et al., 2013; Wang et al., 2017). NAPs can impact chromosome organization by condensing macrodomains, by bridging, by stabilizing supercoils, or by associating with the cell membrane (Amemiya et al., 2021; Dame et al., 2020). Transcription gives rise to twin‐supercoiled‐domains, with positive supercoiling ahead and negative supercoiling behind RNA polymerases (Junier et al., 2023). The insertion of newly translated proteins into the cell membrane (transertion) causes certain genes to associate with the cell wall (Roggiani & Goulian, 2015).

In its essence, a bacterial chromosome is a long polymer confined to a small volume. The simplest physical models for bacterial chromosome organization explore how different, often biologically motivated, forces and constraints affect the polymer's organization and dynamics (Figure 1a).

### 2.1 Geometric constraints

The volume of confinement and constraining loci within the cell are examples of geometric constraints on a bacterial chromosome. Even these minimal constraints can explain well‐known features of bacterial chromosome organization: tethering the origin of replication to a cell pole can give rise to linear chromosome organization (Buenemann & Lenz, 2010, 2011), as seen in species such as *C. crescentus*, while tight confinement can drastically change the way that polymers interact, with implications for bacterial chromosome segregation.

Jun and Mulder (2006) proposed that bacterial chromosome segregation could be explained by entropic forces acting on two confined polymers. Consider two polymers consisting of *N* monomers, confined to a long cylinder of diameter *d*. Since below length scales *d*, each polymer is unaffected by the confinement, we can split each polymer into subsections called “confinement blobs,” inside which the polymer is unconstrained. Each blob is constrained to lie along the long axis of the cylinder, which introduces an entropic cost. The entropic cost of confinement can hence be shown to scale with the number of confinement blobs, $N_{\text{blobs}}∼Nd^{-1⁄\nu }$, where ν is the Flory exponent, describing how much the polymer tends to swell in a given solvent. Since overlap of any two blobs is entropically costly, the two polymers will entropically segregate. Such arguments can be extended for ring polymers and for shorter cylinders (Jun & Wright, 2010; Jung et al., 2012), suggesting that replicated bacterial chromosomes could entropically segregate in cellular confinement.

Despite theoretical arguments for entropic segregation, several simulation studies have shown that, without additional constraints, circular chromosomes do not segregate at intermediate replication stages. Constraints such as the concentric‐shell model (Jun & Mulder, 2006), confining sections of the chromosome to sub‐volumes of the nucleoid (modeling Macrodomains of the *E. coli* chromosome) (Junier et al., 2014), fixing the replisomes at mid‐cell (El Najjar et al., 2020), or linking chromosomal arms with loop‐extruders (Harju et al., 2023) have been necessary to achieve segregation concurrent with replication. Why are such additional constraints needed? We recently argued that at intermediate replication stages, purely entropic forces can actually *inhibit* bacterial chromosome segregation by pushing replication forks apart (Harju et al., 2023). Additionally, free energy calculations have shown that the time delay before entropic segregation begins can grow exponentially with the chain length (Minina & Arnold, 2014, 2015; Polson & Kerry, 2018) and that two polymers of different lengths do not necessarily demix in confinement (Polson & Zhu, 2021). Both due to partially conflicting simulation results and the lack of experimental evidence, the role of entropy in bacterial chromosome organization remains a subject of debate. Furthermore, we still lack theoretical understanding of chromosome segregation in spherically shaped cocci (Pinho et al., 2013), and in species with multiple chromosomes of different topologies (Ren et al., 2022).

### 2.2 Topological constraints

The shape of a bacterial chromosome is characterized by its topology: a linear, a circular, and a partially replicated chromosome all have different numbers of loops (0, 1, or 2) and are hence topologically distinct. We will now discuss how topological changes can affect the organization and dynamics of bacterial chromosomes.

Mitra et al. (2022a, 2022b) recently proposed that fixed loops at the boundaries of *E. coli* Macrodomains (Figure 1a) could be sufficient to explain experimentally observed chromosome organization and segregation patterns. The authors showed that fixed loop architectures give rise to predictable and robust orientation of confined chromosomes, and that excluded volume interactions between loops can enhance chromosome segregation. Although it remains to be shown whether such stable loops at fixed genomic positions are common in bacteria, these findings also suggest that more randomly placed loops could affect the direction of entropic forces at the single‐cell level.

Another example of a topological constraint on a bacterial chromosome is its supercoiling level. To illustrate, consider holding the ends of a piece of ribbon, and twisting them in opposite directions. This causes the ribbon to writhe around its central axis. If you now release tension by bringing the ends of the ribbon closer together, the ribbon will coil up into a plectoneme, but the number of turns (the linking number) will be conserved. Similarly, the supercoiling of bacterial chromosomes by active mechanisms causes the DNA to branch into plectonemes (Dorman, 2019; Junier et al., 2023). This observation motivated the development of “feather‐boa” models, where the chromosome is considered to consist of loops or branches emanating from a backbone (Reviewed in Ha and Jung [2015]).

Branching can both compact chromosomes and enhance their segregation (Jun & Wright, 2010). Additionally, feather‐boa models have been shown to reproduce experimentally observed features of bacterial chromosome organization and dynamics, such as subdiffusive motion of chromosomal loci (Yu et al., 2021), and helical ordering of chromosomal arms (Swain et al., 2019). Feather‐boa models hence remain an active area of research, and new computational advances are improving simulation resolutions and speeds (Ghobadpour et al., 2021; Goodsell et al., 2018). Despite these computational advances, an open‐standing theoretical question is how branches and loops should be (dynamically) distributed in bacterial chromosome models, given what we know about their underlying causes.

## 3 BOTTOM‐UP MODELING

In this section, we focus on bottom‐up models, which model how chromosome organization emerges from proposed biological mechanisms (Figure 1b). Such models can describe how transcription gives rise to plectoneme branches, or how macromolecular crowding confines the nucleoid to only 40%–90% of the cell (Gray et al., 2019).

A strength of bottom‐up models is that they provide mechanistic insight and make novel predictions. A limitation is that more complex aspects of chromosome organization may be affected by several distinct mechanisms acting in unison. To illustrate, recent work by Joyeux (2021, 2023) has shown that crowding and supercoiling can compact the chromosome in non‐additive ways at high supercoiling densities, and that macromolecular crowding can enhance chromosome compaction by crosslinkers. These works illustrate that different bacterial chromosome organization mechanisms do not act in isolation. Despite such challenges, bottom‐up models can provide conceptual insight into how various molecular mechanism control bacterial chromosome organization.

### 3.1 Loop extrusion

**FIGURE 2:**
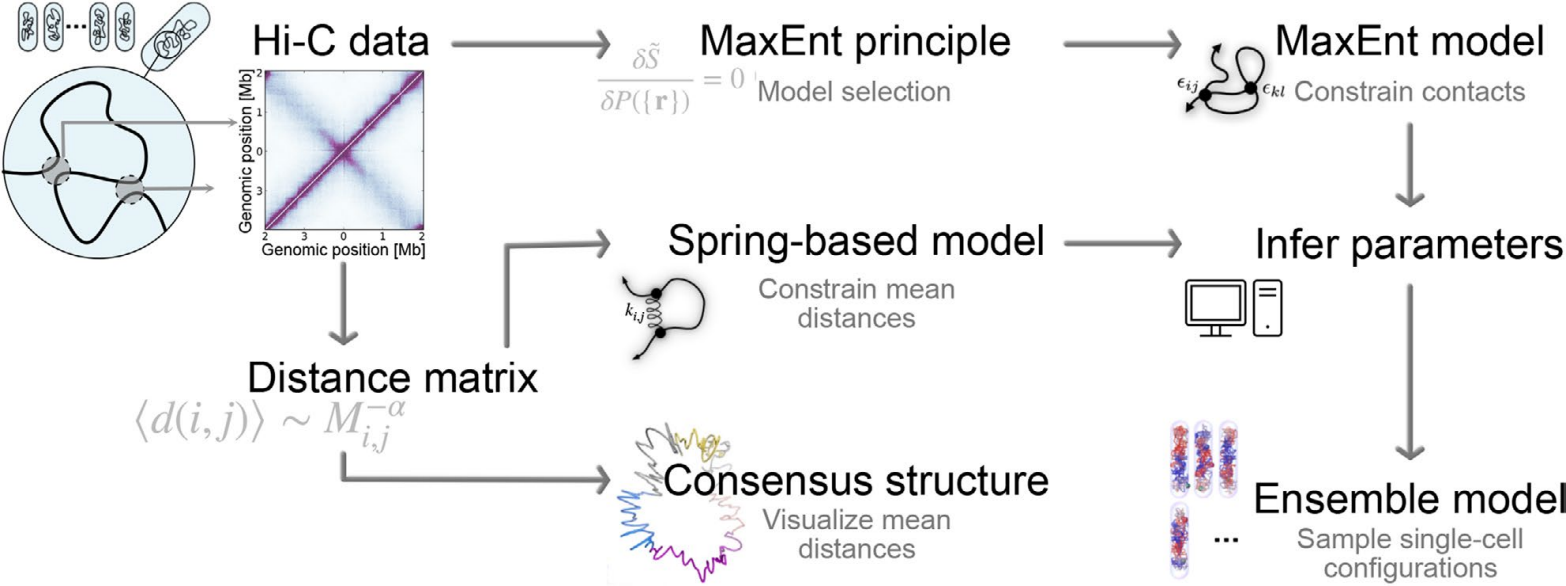
Data‐driven modeling of bacterial chromosomes. Data‐driven models aim to infer a model for bacterial chromosomes from Hi‐C data, which reflects the population‐averaged contact counts between chromosomal regions. Subfigure shows Hi‐C data for *C. crescentus* swarmer cells (Le et al., 2013). Most models start by converting the Hi‐C map to a distance matrix, for instance by assuming a scaling between mean distances between loci $d\left(i,j\right)$ and their contact counts $M_{i,j}.$ Mean distances can be used to create a consensus structure, which depicts the estimated mean distances using a 3D curve. Subfigure shows consensus structure for *E. coli*, adapted from (Hua & Ma, 2019). Alternatively, mean distances can be used to constrain spring‐based ensemble models, with effective harmonic potentials between loci. An ensemble model can also be inferred directly from Hi‐C data: by maximizing the distribution entropy with constraints on contact probabilities $\left(\tilde{S}\right)$, one can choose the least‐assuming chromosome configuration distribution $P_{\text{model}}\left(\left\{r\right\}\right)$ consistent with Hi‐C data (Messelink et al., 2021). The MaxEnt procedure selects a model that features effective close‐range interactions between monomers. For both spring‐based and MaxEnt ensemble models, the effective interaction parameters need to be inferred using computational approaches. Once these parameters have been determined, the distribution can be sampled for single‐cell chromosome configurations. Subfigure shows sampled configurations from the MaxEnt model for *C. crescentus* swarmer cells (Messelink et al., 2021).

Hi‐C experiments, which provide a measure for pairwise contact frequencies between loci, have revealed that bacterial chromosomes are more ordered than homogeneous, randomly oriented polymers in confinement. For instance, bacterial condensin mediates long‐range contacts between the two chromosomal arms in species such as *C. crescentus* (Le et al., 2013), and *B. subtilis* (Wang et al., 2017), resulting in a prominent off‐diagonal trace on Hi‐C maps (Figure 2, top‐left). By contrast, another SMC, MukBEF, enhances long‐range contacts across large parts of the *E. coli* chromosome (Lioy et al., 2018). These findings have triggered the development of loop extrusion models for bacterial chromosomes.

One of the early questions addressed by biophysical modeling of SMCs in bacteria was whether these protein complexes move diffusively or via active loop extrusion. Whereas it was suggested that targeted loading of diffusive slip‐links could be sufficient to model eukaryotic SMC behavior (Brackley et al., 2017) and that MukBEF clustering could arise due to Turing patterning (Murray & Sourjik, 2017), a simulation model for *B. subtilis* (Miermans & Broedersz, 2018) indicated that thousands of diffusive slip‐links were needed to explain off‐diagonal traces on bacterial Hi‐C maps, while only tens of active loop‐extruders were sufficient, more consistent with experimental reports of ~30 condensin complexes per chromosome (Wilhelm et al., 2015). These and other simulations, as well as mounting experimental evidence, have led to loop extrusion becoming more broadly accepted (Banigan & Mirny, 2020; Fudenberg et al., 2017).

MukBEF loop extrusion in *E. coli* has been modeled in 1D (Mäkelä & Sherratt, 2020). The authors proposed that non‐targeted loading of MukBEF gives rise to an array of loops that spans most of the chromosome. Non‐targeted loading of MukBEF could hence result in a feather‐boa structure on parts of the *E. coli* chromosome, reminiscent of microscopy observations of MukBEF distributions in widened *E. coli* cells (Japaridze et al., 2023). However, future work still needs to address how loop extrusion by MukBEF could affect the 3D organization of *E. coli* chromosomes.

The effects of loop extrusion on *B. subtilis* chromosome organization, by contrast, have been modeled by combining 1D loop‐extruder dynamics with 3D polymer simulations (Brandão et al., 2019, 2021). These models better recapitulate Hi‐C data if loop‐extruders slow down as they collide with RNA polymerases at highly transcribed regions. Patterns on Hi‐C maps for strains with two loop‐extruder loading sites, on the other hand, can be explained if loop‐extruders can traverse each other upon collision, as seen in vitro (Kim et al., 2020).

More recently, we modeled how loop‐extruders loaded at the origins of replication affect the segregation and organization of replicating bacterial chromosomes (Harju et al., 2023). This so‐called topo‐entropic segregation model explains how the geometry and effective topology of a replicating chromosome affect the direction of entropic forces. We found that at intermediate replication stages, purely entropic forces inhibit bacterial chromosome segregation. However, loop‐extruders loaded at the origins of replication effectively linearize partially replicated chromosomes, and this change in effective topology redirects entropic forces to enable concurrent replication and segregation.

### 3.2 NAPs and phase separation

In vitro studies have shown that NAPs can locally twist, bend, or bridge DNA (Amemiya et al., 2021; Dame et al., 2020; Song & Loparo, 2015). This shows that NAPs can *locally* affect DNA structure, but what is their impact on bacterial chromosome organization at larger scales? We will first discuss long‐range bridging, which introduces transient cross‐links on bacterial chromosomes. We then turn to liquid–liquid phase separation, which may allow compartmentalization within bacterial cells (Azaldegui et al., 2021; Cohan & Pappu, 2020).

Physically, NAPs can be modeled as particles that can diffuse, interact with each other, and bind to DNA. For bridging to occur, the number of DNA strands that the NAP can simultaneously bind to (its valency) should be at least two. Brackley et al. (2013) showed that, even in the absence of NAP–NAP interactions or cooperative binding, multivalent binding could be sufficient to give rise to NAP clustering. A bivalently binding NAP introduces a loop on the chromosome, which is entropically costly. Two bivalent NAPs can bind far apart, giving rise to two loops, or next to each other, effectively giving rise to just one loop. Hence, even in the absence of NAP–NAP interactions, bridging proteins can cluster for entropic reasons.

Non‐cooperative NAP binding can also affect chromosome dynamics; Subramanian and Murray (2023) showed that transient bridging can give rise to sub‐diffusive motion of loci at timescales below the bridge lifetime. Consistent with this model, an H‐NS mutant of *E. coli* showed weaker sub‐diffusive behavior of loci than the wild type.

Although some NAPs might bind non‐cooperatively, many are known to interact, which can be modeled by introducing NAP–NAP interactions in simulations. Joyeux (2021) studied how protein self‐association impacts chromosome organization. Inspired by the *E. coli* NAP H‐NS, two modes of NAP binding were modeled: filament‐ or cluster‐forming. In simulations, filament‐forming proteins stiffened chromosomal regions where they bound, but did not compact DNA. Conversely, clustering proteins condensed the chromosome, but did not stiffen DNA. This work illustrates that even simple coarse‐grained models can capture a variety of NAP behaviors.

Certain NAP–NAP interactions can give rise to collective phenomena, such as biomolecular condensation. For instance, HU and Dps, two important NAPs in *E. coli*, have been observed to lead to phase separation of DNA segments in vitro (Gupta et al., 2023). Put simply, phase separation can occur when attractive interactions start to dominate over entropic effects; whereas entropy favors spreading NAPs across the accessible volume, attractive NAP–NAP interactions of suitable geometry and sufficient range can favor NAP condensation. Since phase separation can create long‐range order, it can impact chromosome organization at large scales.

Some of the earliest evidence for biomolecular condensation in bacterial cells came from observations of ParB clusters forming at *parS* sites on bacterial chromosomes and plasmids (Broedersz et al., 2014; Jalal & Le, 2020). Bottom‐up models have been used to explore transport (Hu et al., 2017; Köhler & Murray, 2023; Lim et al., 2014; Surovtsev et al., 2016; Walter et al., 2017) and force generation (Hanauer et al., 2021) by the ParAB*S* system, as well as ParB cluster formation (Broedersz et al., 2014; Sanchez et al., 2015; Walter et al., 2021). These works offer two key physical insights. First, as opposed to earlier works that assumed that ParB only spreads along the one‐dimensional DNA strand (Breier & Grossman, 2007; Murray et al., 2006), the formation of ParB clusters is an inherently three‐dimensional phenomenon; a well‐known result from statistical physics states that phase separation cannot occur in one‐dimensional systems with short‐range interactions. Hence 3D bridging and fluctuations of the chromosome are important for the formation of ParB clusters. Accordingly, in vitro experiments have confirmed that bridging is essential for ParB spreading (Graham et al., 2014) and that ParB‐dimers can recruit each other in‐trans and form dynamic clusters via bridging (Tišma et al., 2022, 2023). Second, while phase‐separated droplets at equilibrium should coalesce over time, either via collision of droplets or Ostwald ripening (when constituents diffuse from smaller droplets to larger ones), separate droplets could be maintained under energy‐consuming non‐equilibrium conditions. This suggests that the maintenance of ParB condensates on separate plasmids and/or *parS* sites may require an active mechanism, such as ParA ATPase (Guilhas et al., 2020) and/or ParB CTPase activity.

Recent experiments have revealed that CTP binding allows ParB complexes to first clamp onto and then slide along DNA (Jalal et al., 2020; Osorio‐Valeriano et al., 2019; Soh et al., 2019). Simulations of only diffusive sliding have been shown to recapitulate experimental ChIP‐seq data (Osorio‐Valeriano et al., 2021), suggesting this is a prominent mechanism of ParB spreading. More recent models have combined 1D sliding with 3D bridging mechanisms. Connolley et al. (2023) constructed an implicit model, where pairwise bridges between loci can form with a probability proportional to the ChIP‐seq scores at these sites. Tišma et al. (2023) conducted explicit molecular dynamics simulations of ParB dynamics, assuming 1D sliding and ParB‐ParB interactions, and found that multivalent ParB‐ParB interactions are necessary for the formation of dense clusters, a prerequisite for forming the partition complex.

### 3.3 Effects of transcription

Transcription and translation can affect bacterial chromosome organization in multiple ways. Steric interactions with ribosomes and RNA can affect nucleoid compaction and localization (Miangolarra et al., 2021; Xiang et al., 2021). Transertion—the insertion of membrane proteins into the cell wall as they are translated and transcribed (Roggiani & Goulian, 2015; Spahn et al., 2023)—can cause loci to remain near the cell membrane. Highly transcribed genes have been proposed to colocalize, since RNA polymerases can cluster in fast‐growth conditions (Ladouceur et al., 2020). Finally, transcription introduces both positive and negative supercoils, and highly transcribed genes can act as topological barriers that inhibit plectoneme diffusion (Le et al., 2013; Le & Laub, 2016). Since single‐molecule experiments are providing evidence that some NAPs (Guo et al., 2021) and potentially SMCs (Kim et al., 2022) are recruited to areas of high supercoiling, future models could explore the interplay of these different mechanisms of bacterial chromosome organization.

By comparing Monte Carlo simulations of a confined polymer to experimental data, Xiang et al. (2021) argued that the mesh size of the *E. coli* nucleoid is compatible with the chromosome being embedded in an “effective poor solvent”. Briefly, a polymer in a poor solvent will tend to condense, since monomer‐monomer interactions are energetically more favorable than monomer‐solvent interactions. Since ribosome and DNA densities were found to be anti‐correlated, Xiang et al. (2021) suggested that this effective poor solvent could be a result of excluded volume interactions between the chromosome and ribosomes and/or RNA. However, it remains unclear to what extent the cytoplasm and all its constituents that interact with DNA via both equilibrium and non‐equilibrium processes can be modeled as an effective solvent. Miangolarra et al. (2021) explored steric interactions between bacterial chromosomes and the transcriptional‐translational machinery. By modeling the coupled 1D dynamics of DNA, ribosomes, and mRNA, they showed how active transcription and translation can affect the shape, size, and position of the nucleoid.

As reviewed by Junier et al. (2023), supercoiling due to transcription has not yet been modeled at scales of the bacterial chromosome. This is mainly due to computational limitations: bottom‐up simulations for transcription‐induced supercoiling in 3D have only been conducted for scales of tens of kilobases (Lepage & Junier, 2019). In light of these limitations, recent chromosome‐scale models have considered branched polymers with plectoneme distributions that correlate with transcriptional activity (Hacker et al., 2017; Wasim et al., 2023a, 2023b). To illustrate, Hacker et al. (2017) divided the *E. coli* chromosome into “plectoneme‐rich” and “plectoneme‐free” regions based on RNAP ChIP‐seq data, and then simulated branched polymers with sampled plectoneme configurations. Such use of complex, quantitative experimental data to constrain a model is a defining characteristic of modern data‐driven modeling, which can offer new conceptual and mechanistic insights into bacterial chromosome organization.

## 4 DATA‐DRIVEN MODELS

Over the last decade, Hi‐C experiments have led to a breakthrough in studying chromosome organization quantitatively. A typical Hi‐C map for a bacterial chromosome at a 5–10 kb resolution consists of ~160,000 data points, accurately probing features of chromosome organization over 3 orders of magnitude in genomic scales. Unlike microscopy methods, however, Hi‐C experiments do not yield easily interpretable images, but rather a statistical metric for population‐averaged pairwise contact counts. Using Hi‐C data to faithfully extract information about the underlying distribution of three‐dimensional chromosome configurations is thus a daunting theoretical challenge.

Data‐driven theoretical approaches seek to exploit the quantitative potential of Hi‐C maps by directly inferring a model for 3D chromosome organization from experimental data (Contessoto et al., 2022). Since Hi‐C data represent an ensemble average of contact frequencies over the full distribution $P\left(\left\{r\right\}\right)$ of 3D chromosome conformations $\left\{r\right\}$, data‐driven models for bacterial chromosome organization usually seek to find either a single “average” chromosome consensus structure, ${\left\{r\right\}}_{\text{consensus}}$, or an ensemble of chromosome configurations, $P_{\text{model}}\left(\left\{r\right\}\right)$ (McCord et al., 2020). Inference of both types of models is a technically challenging inverse problem, as we discuss below.

### 4.1 Consensus structure models

Most data‐driven models do not use Hi‐C data directly as input, and consensus structure algorithms are no exception (Figure 2). To construct a consensus structure, Hi‐C scores are first converted into average spatial distances between locus pairs. This can be done by assuming that the mean distance between loci has a power‐law scaling with the contact frequency (Marbouty et al., 2015), or by using an experimentally determined calibration curve (Umbarger et al., 2011). Theoretically, however, the pairwise contact frequency between two foci is expected to depend not only on their mean distance but also on the distance distribution's shape. Accordingly, experimental (Lioy et al., 2018) and simulation (Messelink et al., 2021) results show that mean distances between chromosomal loci can show large deviations from average scalings. Nevertheless, once an average distance map has been found, computational algorithms (reviewed by Liu et al. [2023]) can be used to find a single 3D structure where the pairwise distances between loci are as compatible with the estimated mean distances as possible.

How should we interpret a consensus structure? Unlike proteins that often fold into specific, robust shapes that are critical for their function, bacterial chromosomes are highly flexible and dynamic polymers; imaging experiments show that the positions of chromosomal loci can vary by as much as half a cell length (Viollier et al., 2004). This inherent conformational variability is neglected by consensus structure algorithms: they cannot predict population‐level variations in chromosome organization. Nonetheless, consensus structures may offer intuition for global chromosome organization by providing a “convenient visualization tool” (Lioy et al., 2018) for estimated mean distances between chromosomal regions. Furthermore, comparison of consensus structures for mutant strains or for drug‐treated cells might yield clues about how different perturbations affect global chromosome organization.

Umbarger et al. (2011) applied an algorithm originally developed for macromolecular assemblies such as nuclear pore complexes (Russel et al., 2012) to predict consensus structures for a *C. crescentus* chromosome based on 5C data. A set of candidate structures were found by initializing the algorithm with different initial conditions, and the inferred structures were then grouped by similarity. The model suggested that the arms of the *C. crescentus* chromosome are wound in a loose helical structure. The authors also inferred structures for a mutant where the *parS* site was relocated, leading to a shift in the cross‐diagonal line on the 5C map. The corresponding consensus structure showed that the end of the nucleoid shifted to the new location of the *parS* site, consistent with this site being tethered to a cell pole.

More recently, an error vector resultant algorithm was developed for faster and more accurate inference of consensus structures for prokaryotic chromosomes (Hua & Ma, 2019). The algorithm was applied to Hi‐C data from *C. crescentus*, *E. coli*, and *B. subtilis*. By comparing consensus structures for wild type and a Δfis mutant of *E. coli*, the authors concluded that the terminal region bends towards the rest of the chromosome in the mutant strain, reflecting increased Hi‐C counts between the terminal region and the rest of the chromosome. Contrasting earlier consensus structures (Marbouty et al., 2015; Umbarger et al., 2011), helicity of the arms was only predicted for *B. subtilis*. Such contradictory results raise further questions about how consensus structures relate to the underlying distribution of chromosome configurations in individual cells.

### 4.2 Ensemble models

Ensemble methods aim to capture population‐level variability in bacterial chromosome organization by finding a *distribution* $P_{\text{model}}\left(\left\{r\right\}\right)$ of single‐cell chromosome configurations, given Hi‐C data (Figure 2). Most approaches assume an underlying statistical model for this distribution, defined by a set of effective interaction parameters. Once the effective parameters have been inferred from data, the model ensemble can be sampled using statistical methods such as Monte Carlo simulations. Samples from the distribution can be interpreted as single‐cell chromosome configurations. In this way, an ensemble model constructed using population‐averaged data can be used to make predictions about chromosome organization both on the single‐cell and population level.

Like consensus structure models, most data‐driven ensemble models for bacterial chromosome organization start by assuming a relation between Hi‐C scores and average monomer distances. However, these distances are now typically used to define spring‐like interactions between loci, which constrain the mean distances in the model to match input data. For example, Yildirim and Feig (2018) constructed an ensemble model for the *C. crescentus* chromosome by first converting Hi‐C scores to expected distances between loci based on previous calibration data (Umbarger et al., 2011), and then constraining distances between a subset of monomer pairs in a plectonemic model using spring‐like interactions. Equilibrium molecular dynamics simulations were used to produce an ensemble of chromosome configurations, and configurations were assigned statistical weights based on how closely their distance matrices matched input Hi‐C data. The correlation between the model's contact map and the experimental Hi‐C map (0.88) was comparable to that between Hi‐C maps of *C. crescentus* and *B. subtilis* (0.878 for Hi‐C maps from Le et al. [2013], Wang et al. [2015]). This illustrates that models constrained with inferred distances do not necessarily reproduce the Hi‐C map faithfully.

Using a similar approach, Wasim et al. (2021) constructed a spring‐based model for the *E. coli* chromosome. The authors have since studied the sub‐diffusional behavior of loci in their model, compared models for wild‐type cells and HU‐ and MatP‐mutants, and constructed a model with plectonemes (Bera et al., 2022; Wasim et al., 2023). These works have suggested that locus (sub‐)diffusion depends on genomic position and that inclusion of plectonemes in the model slightly affected chromosome compaction, but not organization.

These and other ensemble techniques have advanced data‐driven modeling beyond consensus structure inference for both pro‐ and eukaryotic chromosomes (Marti‐Renom et al., 2018; McCord et al., 2020; Oluwadare et al., 2019). However, several issues remain. Many ensemble approaches rely on strong assumptions, like thermal equilibrium or converting Hi‐C counts to expected mean distances. Furthermore, the diversity of methods hints at a more fundamental concern: while all these approaches lead to *a* model based on a given Hi‐C map, many distinct ensembles could be consistent with the same data. So, how do you select the right one?

To address this challenge, our group developed a data‐driven model for bacterial chromosome organization based on the Maximum Entropy (MaxEnt) principle. This principle selects the unique chromosome conformation distribution $P_{\text{MaxEnt}}\left(\left\{r\right\}\right)$ that reproduces a given Hi‐C map but is otherwise as unstructured as possible. Notably, the model does not rely on converting Hi‐C scores to mean pairwise distances between loci. We applied this approach to model chromosome organization in newborn *C. crescentus* swarmer cells (Messelink et al., 2021). As validation, we showed that the model accurately predicts the long‐axis distributions of loci over the entire chromosome, as measured by independent experiments (Viollier et al., 2004). The MaxEnt model can also reveal novel features of chromosome organization. For example, our model predicted the presence of “super‐domains”, or clusters of high chromosomal density at the single‐cell level. The presence of these super‐domains was validated using super‐resolution microscopy.

Our MaxEnt model was limited to new‐born cells with a single chromosome, constrained using Hi‐C data from synchronized cells (Le et al., 2013). However, Hi‐C experiments on *E. coli*, *B. subtilis*, and many other bacteria are conducted on asynchronous populations. The resulting Hi‐C maps reflect an average over cells at different replication stages, which poses challenges for data‐driven modeling. For instance, bacterial chromosome organization can vary over the cell cycle (Wang et al., 2014), and Hi‐C experiments in replicating bacteria count both cis‐ and trans‐contacts. Wasim et al. (2021) inferred models for *E. coli* at discrete replication stages by constraining each model with the same asynchronous Hi‐C data, and by assuming that trans‐contacts are negligible. Since the validity of these approximations has yet to be established, an open‐standing question in the field is how to best infer a model using Hi‐C data from an asynchronous population. This is clearly a challenging but worthwhile problem: Such a model could provide new insight into the dynamics of chromosome organization across the cell cycle.

## 5 DISCUSSION AND FUTURE CHALLENGES

We have discussed three biophysical approaches to modeling bacterial chromosomes: models based on imposed geometric or topological constraints, bottom‐up models, and data‐driven approaches. Simple models of (branched) polymers in confinement have revealed how different constraints affect bacterial chromosome organization. Bottom‐up approaches link these constraints to their underlying biophysical causes, and can hence be used to gain mechanistic insights. Data‐driven methods aim to capture detailed chromosome organization by inferring models from experimental Hi‐C data. Importantly, we note that these modeling approaches can complement each other. For instance, data‐driven approaches can help hypothesize simple physical principles of chromosome organization, which can then guide the construction of bottom‐up or constraint‐based models.

Given that simplified constraint‐based and bottom‐up models are tailored to explain only certain aspects of chromosome organization, it can be difficult to test their predictions in vivo with controlled and targeted perturbations. To test whether effects seen in polymer simulations are relevant at biological length‐ and time‐scales, these simplified models could be compared to artificial systems of chromosomes in confinement (Birnie & Dekker, 2021). Conversely, bottom‐up models could be constructed for “simpler” organisms: Stevens et al. (2023) recently presented a model for an entire, minimal bacterial cell, with a 543 kb long circular chromosome. For more complex systems, the increased availability of high‐quality quantitative data creates opportunities for data‐driven modeling. For instance, multiple types of data, such as Hi‐C, imaging, and/or RNA‐sequencing, can be combined to infer data‐driven models that capture bacterial chromosome organization in its full complexity (Messelink et al., 2021; Wasim et al., 2023a).

In conclusion, biophysical modeling has helped shape our understanding of how bacterial chromosomes are functionally organized. Since biophysical models can be easily adapted for different organisms, modeling can help search for divergent and unifying principles of prokaryotic genome organization.

## AUTHOR CONTRIBUTIONS

**Janni Harju:** Investigation; writing – original draft; writing – review and editing; visualization; conceptualization. **Chase P. Broedersz:** Conceptualization; writing – original draft; writing – review and editing; supervision.

## ETHICS STATEMENT

No human or animal subjects or materials were used in this review.

## Data Availability

Data sharing is not applicable to this article as no new data were created or analyzed in this study.

## References

[mmi15257-bib-0001] Amemiya, H.M. , Schroeder, J. & Freddolino, P.L. (2021) Nucleoid‐associated proteins shape chromatin structure and transcriptional regulation across the bacterial kingdom. Transcription, 12(4), 182–218. Available from: 10.1080/21541264.2021.1973865 34499567 PMC8632127

[mmi15257-bib-0002] Azaldegui, C.A. , Vecchiarelli, A.G. & Biteen, J.S. (2021) The emergence of phase separation as an organizing principle in bacteria. Biophysical Journal, 120(7), 1123–1138. Available from: 10.1016/j.bpj.2020.09.023 33186556 PMC8059088

[mmi15257-bib-0003] Banigan, E.J. & Mirny, L.A. (2020) Loop extrusion: theory meets single‐molecule experiments. Current Opinion in Cell Biology, 64, 124–138. Available from: 10.1016/J.CEB.2020.04.011 32534241

[mmi15257-bib-0004] Bera, P. , Wasim, A. & Mondal, J. (2022) Hi‐C embedded polymer model of Escherichia coli reveals the origin of heterogeneous subdiffusion in chromosomal loci. Physical Review E, 105, 064402. Available from: 10.1103/PhysRevE.105.064402 35854496

[mmi15257-bib-0005] Birnie, A. & Dekker, C. (2021) Genome‐in‐a‐box. ACS Nano, 15(1), 111–124. Available from: 10.1021/acsnano.0c07397 33347266 PMC7844827

[mmi15257-bib-0006] Brackley, C.A. , Johnson, J. , Michieletto, D. , Morozov, A.N. , Nicodemi, M. , Cook, P.R. et al. (2017) Nonequilibrium chromosome looping via molecular slip links. Physical Review Letters, 119(13), 138101. Available from: 10.1103/PhysRevLett.119.138101 29341686

[mmi15257-bib-0007] Brackley, C.A. , Taylor, S. , Papantonis, A. , Cook, P.R. & Marenduzzo, D. (2013) Nonspecific bridging‐induced attraction drives clustering of DNA‐binding proteins and genome organization. Proceedings of the National Academy of Sciences of the United States of America, 110(38), E3605–E3611. Available from: 10.1073/pnas.1302950110 24003126 PMC3780866

[mmi15257-bib-0008] Brandão, H.B. , Paul, P. , van den Berg, A.A. , Rudner, D.Z. , Wang, X. & Mirny, L.A. (2019) RNA polymerases as moving barriers to condensin loop extrusion. Proceedings of the National Academy of Sciences of the United States of America, 116(41), 20489–20499. Available from: 10.1073/pnas.1907009116 31548377 PMC6789630

[mmi15257-bib-0009] Brandão, H.B. , Ren, Z. , Karaboja, X. , Mirny, L.A. & Wang, X. (2021) DNA‐loop‐extruding SMC complexes can traverse one another in vivo. Nature Structural & Molecular Biology, 28, 642–651. Available from: 10.1038/s41594-021-00626-1 PMC8878250 34312537

[mmi15257-bib-0010] Breier, A.M. & Grossman, A.D. (2007) Whole‐genome analysis of the chromosome partitioning and sporulation protein Spo0J (ParB) reveals spreading and origin‐distal sites on the Bacillus subtilis chromosome. Molecular Microbiology, 64(3), 703–718. Available from: 10.1111/j.1365-2958.2007.05690.x 17462018

[mmi15257-bib-0011] Broedersz, C.P. , Wang, X. , Meir, Y. , Loparo, J.J. , Rudner, D.Z. & Wingreen, N.S. (2014) Condensation and localization of the partitioning protein ParB on the bacterial chromosome. Proceedings of the National Academy of Sciences of the United States of America, 111(24), 8809–8814. Available from: 10.1073/pnas.1402529111 24927534 PMC4066521

[mmi15257-bib-0012] Buenemann, M. & Lenz, P. (2010) A geometrical model for DNA organization in bacteria. PLoS ONE, 5(11), e13806. Available from: 10.1371/journal.pone.0013806 21085464 PMC2972204

[mmi15257-bib-0013] Buenemann, M. & Lenz, P. (2011) Geometrical ordering of DNA in bacteria. Communicative & Integrative Biology, 4(3), 291–293. 21980561 10.4161/cib.4.3.14891 PMC3187889

[mmi15257-bib-0014] Cohan, M.C. & Pappu, R.V. (2020) Making the case for disordered proteins and biomolecular condensates in bacteria. Trends in Biochemical Sciences, 45(8), 668–680. Available from: 10.1016/j.tibs.2020.04.011 32456986

[mmi15257-bib-0015] Connolley, L. , Schnabel, L. , Thanbichler, M. & Murray, S.M. (2023) Partition complex structure can arise from sliding and bridging of ParB dimers. Nature Communications, 14(4567), 1–12. Available from: 10.1038/s41467-023-40320-y PMC10387095 37516778

[mmi15257-bib-0016] Contessoto, V.G. , Cheng, R.R. & Onuchic, J.N. (2022) Uncovering the statistical physics of 3D chromosomal organization using data‐driven modeling. Current Opinion in Structural Biology, 75, 102418. Available from: 10.1016/J.SBI.2022.102418 35839701

[mmi15257-bib-0017] Dame, R.T. , Rashid, F.Z.M. & Grainger, D.C. (2020) Chromosome organization in bacteria: mechanistic insights into genome structure and function. Nature Reviews Genetics, 21(4), 227–242. Available from: 10.1038/s41576-019-0185-4 31767998

[mmi15257-bib-0018] Dorman, C.J. (2019) DNA supercoiling and transcription in bacteria: a two‐way street. BMC Molecular and Cell Biology, 20(1), 1–9. Available from: 10.1186/s12860-019-0211-6 31319794 PMC6639932

[mmi15257-bib-0019] El Najjar, N. , Geisel, D. , Schmidt, F. , Dersch, S. , Mayer, B. , Hartmann, R. et al. (2020) Chromosome segregation in Bacillus subtilis follows an overall pattern of linear movement and is highly robust against cell cycle perturbations. mSphere, 5(3), e00255‐20. Available from: 10.1128/msphere.00255-20 32554717 PMC7300352

[mmi15257-bib-0020] Fudenberg, G. , Abdennur, N. , Imakaev, M. , Goloborodko, A. & Mirny, L.A. (2017) Emerging evidence of chromosome folding by loop extrusion. Cold Spring Harbor Symposia on Quantitative Biology, 82, 45–55. Available from: 10.1101/sqb.2017.82.034710 29728444 PMC6512960

[mmi15257-bib-0021] Ghobadpour, E. , Kolb, M. , Ejtehadi, M.R. & Everaers, R. (2021) Monte Carlo simulation of a lattice model for the dynamics of randomly branching double‐folded ring polymers. Physical Review E, 104(1), 014501. Available from: 10.1103/PhysRevE.104.014501 34412203

[mmi15257-bib-0022] Gogou, C. , Japaridze, A. & Dekker, C. (2021) Mechanisms for chromosome segregation in bacteria. Frontiers in Microbiology, 12, 685687. Available from: 10.3389/fmicb.2021.685687 34220773 PMC8242196

[mmi15257-bib-0023] Goodsell, D.S. , Autin, L. & Olson, A.J. (2018) Lattice models of bacterial nucleoids. Journal of Physical Chemistry B, 122(21), 5441–5447. Available from: 10.1021/acs.jpcb.7b11770 29338247 PMC5980677

[mmi15257-bib-0024] Graham, T.G.W. , Wang, X. , Song, D. , Etson, C.M. , van Oijen, A.M. , Rudner, D.Z. et al. (2014) ParB spreading requires DNA bridging. Genes & Development, 28(11), 1228–1238. Available from: 10.1101/gad.242206.114 24829297 PMC4052768

[mmi15257-bib-0025] Gray, W.T. , Govers, S.K. , Xiang, Y. , Parry, B.R. , Campos, M. , Kim, S. et al. (2019) Nucleoid size scaling and intracellular organization of translation across bacteria. Cell, 177, 1632. Available from: 10.1016/J.CELL.2019.05.017 31150626 PMC6629263

[mmi15257-bib-0026] Guilhas, B. , Walter, J.‐C. , Rech, J. , David, G. , Walliser, N.O. , Palmeri, J. et al. (2020) ATP‐driven separation of liquid phase condensates in bacteria. Molecular Cell, 79(2), 293–303.e4. Available from: 10.1016/j.molcel.2020.06.034 32679076

[mmi15257-bib-0027] Guo, M.S. , Kawamura, R. , Littlehale, M. , Marko, J.F. & Laub, M.T. (2021) High‐resolution, genome‐wide mapping of positive supercoiling in chromosomes. eLife, 10, 7. Available from: 10.7554/ELIFE.67236 PMC8360656 34279217

[mmi15257-bib-0028] Gupta, A. , Joshi, A. , Arora, K. , Mukhopadhyay, S. & Guptasarma, P. (2023) The bacterial nucleoid‐associated proteins, HU and Dps, condense DNA into context‐dependent biphasic or multiphasic complex coacervates. The Journal of Biological Chemistry, 299(5), 104637. Available from: 10.1016/j.jbc.2023.104637 36963493 PMC10141540

[mmi15257-bib-0029] Ha, B.Y. & Jung, Y. (2015) Polymers under confinement: single polymers, how they interact, and as model chromosomes. Soft Matter, 11, 2333–2352. Available from: 10.1039/C4SM02734E 25710099

[mmi15257-bib-0030] Hacker, W.C. , Li, S. & Elcock, A.H. (2017) Features of genomic organization in a nucleotide‐resolution molecular model of the *Escherichia coli* chromosome. Nucleic Acids Research, 45(13), 7541–7554. Available from: 10.1093/nar/gkx541 28645155 PMC5570083

[mmi15257-bib-0031] Hanauer, C. , Bergeler, S. , Frey, E. & Broedersz, C.P. (2021) Theory of active intracellular transport by DNA relaying. Physical Review Letters, 127(13), 138101. Available from: 10.1103/PhysRevLett.127.138101 34623846

[mmi15257-bib-0032] Harju, J. , van Teeseling, M.C.F. & Broedersz, C.P. (2023) Loop‐extruders alter bacterial chromosome topology to direct entropic forces for segregation. *bioRxiv* 10.1101/2023.06.30.547230.2023.06.30.547230 PMC11139863 38816445

[mmi15257-bib-0033] Hu, L. , Anthony, G. , Vecchiarelli, K.M. , Neuman, K.C. & Liu, J. (2017) Brownian ratchet mechanism for faithful segregation of low‐copy‐number plasmids. Biophysical Journal, 112(7), 1489–1502. Available from: 10.1016/j.bpj.2017.02.039 28402891 PMC5390091

[mmi15257-bib-0034] Hua, K.‐J. & Ma, B.‐G. (2019) EVR: reconstruction of bacterial chromosome 3D structure models using error‐vector resultant algorithm. BMC Genomics, 20(1), 738. Available from: 10.1186/s12864-019-6096-0 31615397 PMC6794827

[mmi15257-bib-0035] Jalal, A.S.B. & Le, T.B.K. (2020) Bacterial chromosome segregation by the ParABS system. Open Biology, 10(6), 200097. Available from: 10.1098/rsob.200097 32543349 PMC7333895

[mmi15257-bib-0036] Jalal, A.S.B. , Tran, N.T. & Le, T.B.K. (2020) ParB spreading on DNA requires cytidine triphosphate in vitro. eLife, 9, e53515. Available from: 10.7554/eLife.53515 32077854 PMC7053999

[mmi15257-bib-0037] Japaridze, A. , van Wee, R. , Gogou, C. , Kerssemakers, J.W.J. , van den Berg, D.F. & Dekker, C. (2023) MukBEF‐dependent chromosomal organization in widened *Escherichia coli* . Frontiers in Microbiology, 14, 1107093. Available from: 10.3389/fmicb.2023.1107093 36937278 PMC10020239

[mmi15257-bib-0038] Joyeux, M. (2021) Impact of self‐association on the architectural properties of bacterial nucleoid proteins. Biophysical Journal, 120(2), 370–378. Available from: 10.1016/j.bpj.2020.12.006 33340542 PMC7840413

[mmi15257-bib-0039] Joyeux, M. (2023) Organization of the bacterial nucleoid by DNA‐bridging proteins and globular crowders. Frontiers in Microbiology, 14, 1116776. Available from: 10.3389/fmicb.2023.1116776 36925468 PMC10011147

[mmi15257-bib-0040] Jun, S. & Mulder, B. (2006) Entropy‐driven spatial organization of highly confined polymers: lessons for the bacterial chromosome. Proceedings of the National Academy of Sciences of the United States of America, 103(33), 12388–12393. Available from: 10.1073/pnas.0605305103 16885211 PMC1525299

[mmi15257-bib-0041] Jun, S. & Wright, A. (2010) Entropy as the driver of chromosome segregation. Nature Reviews Microbiology, 8, 600–607. Available from: 10.1038/nrmicro2391 20634810 PMC3148256

[mmi15257-bib-0042] Jung, Y. , Jeon, C. , Kim, J. , Jeong, H. , Jun, S. & Ha, B.‐Y. (2012) Ring polymers as model bacterial chromosomes: confinement, chain topology, single chain statistics, and how they interact. Soft Matter, 8(7), 2095–2102. Available from: 10.1039/C1SM05706E

[mmi15257-bib-0043] Junier, I. , Boccard, F. & Espéli, O. (2014) Polymer modeling of the *E. coli* genome reveals the involvement of locus positioning and macrodomain structuring for the control of chromosome conformation and segregation. Nucleic Acids Research, 42(3), 1461–1473. Available from: 10.1093/nar/gkt1005 24194594 PMC3919569

[mmi15257-bib-0044] Junier, I. , Ghobadpour, E. , Espeli, O. & Everaers, R. (2023) DNA supercoiling in bacteria: state of play and challenges from a viewpoint of physics based modeling. Frontiers in Microbiology, 14, 1192831. Available from: 10.3389/fmicb.2023.1192831 37965550 PMC10642903

[mmi15257-bib-0045] Kim, E. , Gonzalez, A.M. , Pradhan, B. , van der Torre, J. & Dekker, C. (2022) Condensin‐driven loop extrusion on supercoiled DNA. Nature Structural & Molecular Biology, 29, 719–727. Available from: 10.1038/s41594-022-00802-x 35835864

[mmi15257-bib-0046] Kim, E. , Kerssemakers, J. , Shaltiel, I.A. , Haering, C.H. & Dekker, C. (2020) DNA‐loop extruding condensin complexes can traverse one another. Nature, 579, 438–442. Available from: 10.1038/s41586-020-2067-5 32132705

[mmi15257-bib-0047] Köhler, R. & Murray, S.M. (2023) Putting the par back into ParABS: plasmid partitioning driven by ParA oscillations. *bioRxiv* 10.1101/2023.10.16.562490.2023.10.16.562490

[mmi15257-bib-0048] Ladouceur, A.‐M. , Parmar, B.S. , Biedzinski, S. , James Wall, S. , Tope, G. , Cohn, D. et al. (2020) Clusters of bacterial RNA polymerase are biomolecular condensates that assemble through liquid–liquid phase separation. Proceedings of the National Academy of Sciences of the United States of America, 117(31), 18540–18549. Available from: 10.1073/pnas.2005019117 32675239 PMC7414142

[mmi15257-bib-0049] Le, T.B.K. , Imakaev, M.V. , Mirny, L.A. & Laub, M.T. (2013) High‐resolution mapping of the spatial organization of a bacterial chromosome. Science, 342(6159), 731–734. Available from: 10.1126/science.1242059 24158908 PMC3927313

[mmi15257-bib-0050] Le, T.B.K. & Laub, M.T. (2016) Transcription rate and transcript length drive formation of chromosomal interaction domain boundaries. The EMBO Journal, 35, 1582–1595. Available from: 10.15252/EMBJ.201593561 27288403 PMC4946140

[mmi15257-bib-0051] Lepage, T. & Junier, I. (2019. ISSN 0378‐4371) A polymer model of bacterial supercoiled DNA including structural transitions of the double helix. Physica A, 527, 121196. Available from: 10.1016/j.physa.2019.121196

[mmi15257-bib-0052] Lim, H.C. , Surovtsev, I.V. , Beltran, B.G. , Huang, F. , Bewersdorf, J. & Jacobs‐Wagner, C. (2014) Evidence for a DNA‐relay mechanism in ParABS‐mediated chromosome segregation. eLife, 3, e02758. Available from: 10.7554/eLife.02758 24859756 PMC4067530

[mmi15257-bib-0053] Lioy, V.S. , Cournac, A. , Marbouty, M. , Duigou, S. , Mozziconacci, J. , Espéli, O. et al. (2018) Multiscale structuring of the *E. coli* chromosome by nucleoid‐associated and condensin proteins. Cell, 172(4), 771–783.e18. Available from: 10.1016/j.cell.2017.12.027 29358050

[mmi15257-bib-0054] Lioy, V.S. , Junier, I. & Boccard, F. (2021) Multiscale dynamic structuring of bacterial chromosomes. Annual Review of Microbiology, 75(1), 541–561. Available from: 10.1146/annurev-micro-033021-113232 34343019

[mmi15257-bib-0055] Liu, T. , Qiu, Q.‐T. , Hua, K.‐J. & Ma, B.‐G. (2023) Evaluation of chromosome structure modelling tools in bacteria. *bioRxiv*, 2023.10.26.564237.

[mmi15257-bib-0056] Mäkelä, J. & Sherratt, D.J. (2020) Organization of the *Escherichia coli* chromosome by a MukBEF axial core. Molecular Cell, 78, 250–260.e5. Available from: 10.1016/J.MOLCEL.2020.02.003 32097603 PMC7163298

[mmi15257-bib-0057] Marbouty, M. , Le Gall, A. , Cattoni, D.I. , Cournac, A. , Koh, A. , Fiche, J.B. et al. (2015) Condensin‐ and replication‐mediated bacterial chromosome folding and origin condensation revealed by hi‐C and super‐resolution imaging. Molecular Cell, 59, 588–602. Available from: 10.1016/J.MOLCEL.2015.07.020 26295962

[mmi15257-bib-0058] Marti‐Renom, M.A. , Almouzni, G. , Bickmore, W.A. , Bystricky, K. , Cavalli, G. , Fraser, P. et al. (2018) Challenges and guidelines toward 4D nucleome data and model standards. Nature Genetics, 50, 1352–1358. Available from: 10.1038/s41588-018-0236-3 30262815

[mmi15257-bib-0059] McCord, R.P. , Kaplan, N. & Giorgetti, L. (2020) Chromosome conformation capture and beyond: toward an integrative view of chromosome structure and function. Molecular Cell, 77, 688–708. Available from: 10.1016/J.MOLCEL.2019.12.021 32001106 PMC7134573

[mmi15257-bib-0060] Messelink, J.J.B. , van Teeseling, M.C.F. , Janssen, J. , Thanbichler, M. & Broedersz, C.P. (2021) Learning the distribution of single‐cell chromosome conformations in bacteria reveals emergent order across genomic scales. Nature Communications, 12, 1–9. Available from: 10.1038/s41467-021-22189-x PMC8010069 33785756

[mmi15257-bib-0061] Miangolarra, A.M. , Li, S.H.‐J. , Joanny, J.‐F. , Wingreen, N.S. & Castellana, M. (2021) Steric interactions and out‐of‐equilibrium processes control the internal organization of bacteria. Proceedings of the National Academy of Sciences of the United States of America, 118(43), e2106014118. Available from: 10.1073/pnas.2106014118 34675077 PMC8639350

[mmi15257-bib-0062] Miermans, C.A. & Broedersz, C.P. (2018) Bacterial chromosome organization by collective dynamics of SMC condensins. Journal of the Royal Society Interface, 15, 10. Available from: 10.1098/RSIF.2018.0495 PMC6228497 30333247

[mmi15257-bib-0063] Minina, E. & Arnold, A. (2014) Induction of entropic segregation: the first step is the hardest. Soft Matter, 10(31), 5836–5841. Available from: 10.1039/C4SM00286E 24974935

[mmi15257-bib-0064] Minina, E. & Arnold, A. (2015) Entropic segregation of ring polymers in cylindrical confinement. Macromolecules, 48(14), 4998–5005. Available from: 10.1021/acs.macromol.5b00636

[mmi15257-bib-0065] Mitra, D. , Pande, S. & Chatterji, A. (2022a) Polymer architecture orchestrates the segregation and spatial organization of replicating *E. coli* chromosomes in slow growth. Soft Matter, 18(30), 5615–5631. Available from: 10.1039/D2SM00734G 35861071

[mmi15257-bib-0066] Mitra, D. , Pande, S. & Chatterji, A. (2022b) Topology‐driven spatial organization of ring polymers under confinement. Physical Review E, 106(5), 054502. Available from: 10.1103/PhysRevE.106.054502 36559479

[mmi15257-bib-0067] Murray, H. , Ferreira, H. & Errington, J. (2006) The bacterial chromosome segregation protein Spo0J spreads along DNA from parS nucleation sites. Molecular Microbiology, 61(5), 1352–1361. Available from: 10.1111/j.1365-2958.2006.05316.x 16925562

[mmi15257-bib-0068] Murray, S.M. & Sourjik, V. (2017) Self‐organization and positioning of bacterial protein clusters. Nature Physics, 13, 1006–1013.

[mmi15257-bib-0069] Oluwadare, O. , Highsmith, M. & Cheng, J. (2019) An overview of methods for reconstructing 3‐D chromosome and genome structures from hi‐C data. Biological Procedures, 21(1), 1–20. Available from: 10.1186/s12575-019-0094-0 PMC6482566 31049033

[mmi15257-bib-0070] Osorio‐Valeriano, M. , Altegoer, F. , Das, C.K. , Steinchen, W. , Panis, G. , Connolley, L. et al. (2021) The CTPase activity of ParB determines the size and dynamics of prokaryotic DNA partition complexes. Molecular Cell, 81(19), 3992–4007.e10. Available from: 10.1016/j.molcel.2021.09.004 34562373

[mmi15257-bib-0071] Osorio‐Valeriano, M. , Altegoer, F. , Steinchen, W. , Urban, S. , Liu, Y. , Bange, G. et al. (2019) ParB‐type DNA segregation proteins are CTP‐dependent molecular switches. Cell, 179(7), 1512–1524.e15. Available from: 10.1016/j.cell.2019.11.015 31835030

[mmi15257-bib-0072] Pinho, M.G. , Kjos, M. & Veening, J.W. (2013) How to get (a)round: mechanisms controlling growth and division of coccoid bacteria. Nature Reviews Microbiology, 11(9), 601–614. Available from: 10.1038/nrmicro3088 23949602

[mmi15257-bib-0073] Polson, J.M. & Kerry, D.R.‐M. (2018) Segregation of polymers under cylindrical confinement: effects of polymer topology and crowding. Soft Matter, 14(30), 6360–6373. Available from: 10.1039/C8SM01062E 30028460

[mmi15257-bib-0074] Polson, J.M. & Zhu, Q. (2021) Free energy and segregation dynamics of two channel‐confined polymers of different lengths. Physical Review E, 103(1), 012501. Available from: 10.1103/PhysRevE.103.012501 33601524

[mmi15257-bib-0075] Ren, Z. , Liao, Q. , Karaboja, X. , Barton, I.S. , Schantz, E.G. , Mejia‐Santana, A. et al. (2022) Conformation and dynamic interactions of the multipartite genome in *Agrobacterium tumefaciens* . Proceedings of the National Academy of Sciences of the United States of America, 119(6), e2115854119. Available from: 10.1073/pnas.2115854119 35101983 PMC8833148

[mmi15257-bib-0076] Rivas, G. & Minton, A.P. (2016) Macromolecular crowding in vitro, in vivo, and in between. Trends in Biochemical Sciences, 41(11), 970–981. Available from: 10.1016/j.tibs.2016.08.013 27669651 PMC5804487

[mmi15257-bib-0077] Roggiani, M. & Goulian, M. (2015) Chromosome‐membrane interactions in bacteria. Annual Review of Genetics, 49(1), 115–129. Available from: 10.1146/annurev-genet-112414-054958 26436460

[mmi15257-bib-0078] Russel, D. , Lasker, K. , Webb, B. , Velázquez‐Muriel, J. , Tjioe, E. , Schneidman‐Duhovny, D. et al. (2012) Putting the pieces together: integrative modeling platform software for structure determination of macromolecular assemblies. PLoS Biology, 10(1), e1001244. Available from: 10.1371/journal.pbio.1001244 22272186 PMC3260315

[mmi15257-bib-0079] Sanchez, A. , Cattoni, D.I. , Walter, J.‐C. , Rech, J. , Parmeggiani, A. , Nollmann, M. et al. (2015) Stochastic self‐assembly of ParB proteins builds the bacterial DNA segregation apparatus. Cell Systems, 1(2), 163–173. Available from: 10.1016/j.cels.2015.07.013 27135801

[mmi15257-bib-0080] Soh, Y.‐M. , Davidson, I.F. , Zamuner, S. , Basquin, J. , Bock, F.P. , Taschner, M. et al. (2019) Self‐organization of parS centromeres by the ParB CTP hydrolase. Science, 366(6469), 1129–1133. Available from: 10.1126/science.aay3965 31649139 PMC6927813

[mmi15257-bib-0081] Song, D. & Loparo, J.J. (2015) Building bridges within the bacterial chromosome. Trends in Genetics, 31, 164–173. Available from: 10.1016/J.TIG.2015.01.003 25682183

[mmi15257-bib-0082] Spahn, C. , Middlemiss, S. , Mariscal, E.G.‐d. , Henriques, R. , Bode, H.B. , Holden, S. et al. (2023) Transertion and cell geometry organize the *Escherichia coli* nucleoid during rapid growth. *bioRxiv*, 2023.10.16.562172.

[mmi15257-bib-0083] Stevens, J.A. , Grünewald, F. , van Tilburg, P.A.M. , König, M. , Gilbert, B.R. , Brier, T.A. et al. (2023) Molecular dynamics simulation of an entire cell. Frontiers in Chemistry, 11, 1106495. Available from: 10.3389/fchem.2023.1106495 36742032 PMC9889929

[mmi15257-bib-0084] Subramanian, S. & Murray, S.M. (2023) Subdiffusive movement of chromosomal loci in bacteria explained by DNA bridging. Physical Review Research, 5(2), 023034. Available from: 10.1103/PhysRevResearch.5.023034

[mmi15257-bib-0085] Surovtsev, I.V. , Campos, M. & Jacobs‐Wagner, C. (2016) DNA‐relay mechanism is sufficient to explain ParA‐dependent intracellular transport and patterning of single and multiple cargos. Proceedings of the National Academy of Sciences of the United States of America, 113(46), E7268–E7276. Available from: 10.1073/pnas.1616118113 27799522 PMC5135302

[mmi15257-bib-0086] Swain, P. , Mulder, B.M. & Chaudhuri, D. (2019) Confinement and crowding control the morphology and dynamics of a model bacterial chromosome. Soft Matter, 15, 2677–2687. Available from: 10.1039/C8SM02092B 30830139

[mmi15257-bib-0087] Tišma, M. , Janissen, R. , Antar, H. , Martin‐Gonzalez, A. , Barth, R. , Beekman, T. et al. (2023) Dynamic ParB–DNA interactions initiate and maintain a partition condensate for bacterial chromosome segregation. Nucleic Acids Research, 51(21), 11856–11875. Available from: 10.1093/nar/gkad868 37850647 PMC10681803

[mmi15257-bib-0088] Tišma, M. , Panoukidou, M. , Antar, H. , Soh, Y.‐M. , Barth, R. , Pradhan, B. et al. (2022) ParB proteins can bypass DNA‐bound roadblocks via dimer‐dimer recruitment. Science Advances, 8(26), eabn3299. Available from: 10.1126/sciadv.abn3299 35767606 PMC9242446

[mmi15257-bib-0089] Umbarger, M.A. , Toro, E. , Wright, M.A. , Porreca, G.J. , Baù, D. , Hong, S.‐H. et al. (2011) The three‐dimensional architecture of a bacterial genome and its alteration by genetic perturbation. Molecular Cell, 44(2), 252–264. Available from: 10.1016/j.molcel.2011.09.010 22017872 PMC3874842

[mmi15257-bib-0090] Viollier, P.H. , Thanbichler, M. , McGrath, P.T. , West, L. , Meewan, M. , McAdams, H.H. et al. (2004) Rapid and sequential movement of individual chromosomal loci to specific subcellular locations during bacterial DNA replication. Proceedings of the National Academy of Sciences of the United States of America, 101(25), 9257–9262. Available from: 10.1073/pnas.0402606101 15178755 PMC438963

[mmi15257-bib-0091] Walter, J.‐C. , Dorignac, J. , Lorman, V. , Rech, J. , Bouet, J.‐Y. , Nollmann, M. et al. (2017) Surfing on protein waves: proteophoresis as a mechanism for bacterial genome partitioning. Physical Review Letters, 119(2), 028101. Available from: 10.1103/PhysRevLett.119.028101 28753349

[mmi15257-bib-0092] Walter, J.‐C. , Lepage, T. , Dorignac, J. , Geniet, F. , Parmeggiani, A. , Palmeri, J. et al. (2021) Supercoiled DNA and non‐equilibrium formation of protein complexes: a quantitative model of the nucleoprotein ParBS partition complex. PLoS Computational Biology, 17(4), e1008869. Available from: 10.1371/journal.pcbi.1008869 33861734 PMC8092679

[mmi15257-bib-0093] Wang, X. , Brandão, H.B. , Le, T.B.K. , Laub, M.T. & Rudner, D.Z. (2017) Bacillus subtilis SMC complexes juxtapose chromosome arms as they travel from origin to terminus. Science, 355(6324), 524–527. Available from: 10.1126/science.aai8982 28154080 PMC5484144

[mmi15257-bib-0094] Wang, X. , Le, T.B.K. , Lajoie, B.R. , Dekker, J. , Laub, M.T. & Rudner, D.Z. (2015) Condensin promotes the juxtaposition of DNA flanking its loading site in Bacillus subtilis. Genes & Development, 29, 1661–1675. Available from: 10.1101/GAD.265876.115 26253537 PMC4536313

[mmi15257-bib-0095] Wang, X. , Llopis, P.M. & Rudner, D.Z. (2014) Bacillus subtilis chromosome organization oscillates between two distinct patterns. Proceedings of the National Academy of Sciences of the United States of America, 111(35), 12877–12882. Available from: 10.1073/pnas.1407461111 25071173 PMC4156703

[mmi15257-bib-0096] Wasim, A. , Bera, P. & Mondal, J. (2023a) Development of a data‐driven integrative model of a bacterial chromosome. Journal of Chemical Theory and Computation, 20, 1673–1688. Available from: 10.1021/acs.jctc.3c00118 37083406

[mmi15257-bib-0097] Wasim, A. , Bera, P. & Mondal, J. (2023b) On the spatial positioning of ribosomes around chromosome in *E. coli* cytoplasm. *bioRxiv*, 2023.07.04.547709. 10.1021/acs.jpcb.4c01210 38560890

[mmi15257-bib-0098] Wasim, A. , Gupta, A. , Bera, P. & Mondal, J. (2023) Interpretation of organizational role of proteins on *E. coli* nucleoid via Hi‐C integrated model. Biophysical Journal, 122, 63–81. Available from: 10.1016/J.BPJ.2022.11.2938 36435970 PMC9822802

[mmi15257-bib-0099] Wasim, A. , Gupta, A. & Mondal, J. (2021) A Hi–C data‐integrated model elucidates E. coli chromosome's multiscale organization at various replication stages. Nucleic Acids Research, 49, 3077–3091. Available from: 10.1093/NAR/GKAB094 33660781 PMC8034658

[mmi15257-bib-0100] Wilhelm, L. , Bürmann, F. , Minnen, A. , Shin, H.‐C. , Toseland, C.P. , Byung‐Ha, O. et al. (2015) SMC condensin entraps chromosomal DNA by an ATP hydrolysis dependent loading mechanism in *Bacillus subtilis* . eLife, 4, e06659. Available from: 10.7554/eLife.06659 25951515 PMC4442127

[mmi15257-bib-0101] Xiang, Y. , Surovtsev, I.V. , Chang, Y. , Govers, S.K. , Parry, B.R. , Liu, J. et al. (2021) Interconnecting solvent quality, transcription, and chromosome folding in *Escherichia coli* . Cell, 184(14), 3626–3642.e14. Available from: 10.1016/j.cell.2021.05.037 34186018

[mmi15257-bib-0102] Yáñez‐Cuna, F.O. & Koszul, R. (2023) Insights in bacterial genome folding. Current Opinion in Structural Biology, 82, 102679. Available from: 10.1016/j.sbi.2023.102679 37604045

[mmi15257-bib-0103] Yildirim, A. & Feig, M. (2018) High‐resolution 3D models of Caulobacter crescentus chromosome reveal genome structural variability and organization. Nucleic Acids Research, 46(8), 3937–3952. Available from: 10.1093/nar/gky141 29529244 PMC5934669

[mmi15257-bib-0104] Yu, S. , Wu, J. , Meng, X. , Chu, R. , Li, X. & Wu, G. (2021) Mesoscale simulation of bacterial chromosome and cytoplasmic nanoparticles in confinement. Entropy, 23, 542. Available from: 10.3390/E23050542 33924872 PMC8146307

